# Effective Induction of Fertile Gametes in Oriental × Trumpet (OT) Lily by High Temperatures

**DOI:** 10.3390/plants12071563

**Published:** 2023-04-05

**Authors:** Yi-Xuan Huang, Peng-Cheng Yu, Qian Zhang, Zhi-Yi Yue, Mei-Ling Piao, Xue Gao, Gui-Xia Jia

**Affiliations:** 1 Beijing Key Laboratory of Ornamental Plants Germplasm Innovation & Molecular Breeding, National Engineering Research Center for Floriculture, Beijing Laboratory of Urban and Rural Ecological Environment, Key Laboratory of Genetics and Breeding in Forest Trees and Ornamental Plants of Ministry of Education, School of Landscape Architecture, Beijing Forestry University, Beijing 100083, China; 2 College of Forestry, Shanxi Agricultural University, Jinzhong 030801, China

**Keywords:** high temperature, fertility restore, meiosis, *Lilium*

## Abstract

Lily is a very important bulb crop, and interspecific distant hybridization is a crucial method of lily breeding. However, F_1_ interspecific hybrids tend to be highly sterile due to low levels of genetic homozygosity. This can be addressed by meiotic polyploidization, which has the advantage of reducing breeding time and being able to promote genetic recombination resulting in many variant progenies. High temperatures have been proven to induce 2n gametes via hindering a spindle formation in several plants, but little has been reported in lilies. In the present study, after observing the correlation between the development of the pollen mother cells (PMCs) and the length of the buds, 28–31 mm long buds were selected as the experimental material, which were at the stage of prophase I–metaphase I. Individual buds were induced at different temperatures (40 °C, 42 °C, and 44 °C) and durations (4 h and 6 h) using self-made multiwire heating equipment, and successfully induced fertile male gametes.. The best results were achieved with treatment of 42 °C for 4 h, reaching a maximum fertile pollen induction rate of 36.64%, while bud mortality was 40%. Two chemicals, colchicine and oryzalin, were also used by injection, and only the treatment with oryzalin obtained fertile gametes, with the highest fertile gamete rate of 15.39% at a concentration of 0.005%, while the bud mortality was 36.67%. This suggests that high temperatures have a superior effect on lily 2n gamete induction. In addition, the pollen obtained from the 6 h induction of high temperature was significantly larger than that from the 4 h induction, with an average diameter of 138.64 μm and 107.88 μm, respectively, 2.35 and 1.84 times wider than haploid pollen. The fertile pollen was crossed with four cultivars and two species, and a total of 267 embryonic seeds were obtained, with the highest embryonic rate of 4.52% in OT lily ‘Mister Cas’ as the parent, which had a germination rate of 26.27%. This suggests that the method of high-temperature induction for fertile gametes probably has important significance for ploidy and distant hybrid breeding in lilies.

## 1. Introduction

*Lilium* consists of about 100 species and can be divided into seven sections [1]. Interspecific distant hybridization is an important tool and breeding trend for breeding new varieties of lilies. While the F_1_ distant hybrids are normally shown as intermediate phenotypic characteristics of both parents and as intermediate breeding material, further backcrossing is required to obtain new cultivars with a superior combination of traits [2]. However, the majority of the F_1_ distant hybrids are highly sterile due to the large distance in genetic composition, and the failure of chromosomes to pair and segregate properly during meiosis leads to the formation of gametes with an unbalanced chromosome constitution, which has largely limited the further improvement and introgression between lily groups [3]. Therefore, restoring the fertility of the F_1_ distant hybrids has become a bottleneck in breeding new cultivars of high-quality distant hybrid lilies.

The most widely used method to restore the fertility of the F_1_ distant hybrids is mitotic polyploidization, which means doubling the number of chromosomes in F_1_ to produce an allopolyploid. The problem of this approach, however, is that the plants with doubled chromosome numbers grow more slowly; moreover, the genetic material is fixed, and a homologous recombination hardly occurs, leading to a long breeding cycle and less variation in the backcross progeny [4]. Another method is meiotic polyploidization, which means inducing PMCs to produce unreduced gametes (2n gametes). This method promotes the occurrence of intergenomic recombination under suitable conditions, so as to produce fertile pollen with rich genetic composition and make the progeny appear more varied, from which the new cultivars with superior comprehensive traits can be screened [5]. At present, most interspecific cultivars of lilies are derived from the 2n gametes of F_1_ hybrids [2].

OT hybrids have gained popularity in recent years due to the excellent resistance and wide range of flower colors. Most of the OT hybrids are triploid and originate from 2n gametes produced by F_1_ distant hybrids. However, OT hybrids are highly male-sterile, and there are very few lily F_1_ distant hybrids that can produce functional 2n pollen under natural conditions to meet breeding needs [6,7,8]. Thus, inducing 2n pollen to restore the lily F_1_ distant hybrid’s fertility and using it in crosses is an effective way to breed new OT lily cultivars because the first hybrid between Trumpet lily and Oriental lily, ‘Black Beauty’ combines the characteristics of both major commercial lilies [9]. However, as a heterozygous diploid, both male and female gametes are highly sterile as the chromosomes do not pair and segregate properly during meiosis, and the restoration of its fertility is therefore of great importance for OT lily breeding.

In previous reports, 2n pollen induction by the injection of colchicine into lily buds was the most commonly used method [10], and oryzalin, as an alternative to colchicine, has gradually gained widespread use in recent years in polyploid breeding efforts [11,12]. Nitrous oxide gas (N_2_O) treatment of buds can also induce 2n pollen [13], while high-temperature treatment, which is less toxic and dangerous, has been proven to induce 2n gametes successfully in *Populus tomentosa* [14], *Hevea brasiliensis* [15], and other plants. However, this has been sporadically reported and can only be achieved by growing lilies in phytotron or heated greenhouses [16,17]. The former had problems of not being able to control the temperature with precision and constancy, whereas the latter was difficult to manipulate for tall plants with inconsistent bud development. In addition, most species and cultivars of lilies prefer cool environments and are poor in heat resistance, showing optimal growth between 15 and 25 °C [18]; once the temperature gets too high, plant growth and development are severely inhibited, which greatly increases the difficulty of inducing lily 2n pollen by heating the whole plant. Based on the current state of exploration of high-temperature treatment in lilies, we developed a piece of multiwire heating equipment (unpublished) to treat individual buds separately to explore the approximate treatment temperature and duration suitable for the induction of 2n gametes in lilies. In the present study, the high-temperature induction of 2n gametes was shown to be more efficient than traditional chemical induction methods and reduced costs, toxicity, and danger. The fertile pollen produced by induction was crossed to obtain embryonic seeds, proving that high-temperature induction is worth promoting in the field of distant hybrid breeding and polyploid breeding in lilies.

## 2. Results

### 2.1. Determination of Meiotic Stage of PMCs in Buds of Different Sizes

**Figure 1:**
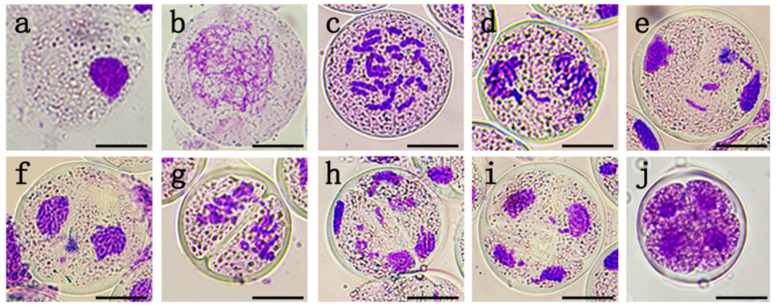
Meiotic processes of PMCs of ‘Black Beauty’. (**a**) Interphase. (**b**) Prophase I. (**c**) Metaphase I. (**d**) Anaphase I. (**e**) Telophase I. (**f**) Prophase II. (**g**) Metaphase II. (**h**) Anaphase II. (**i**) Telophase II. (**j**) Tetrad.

**Figure 2:**
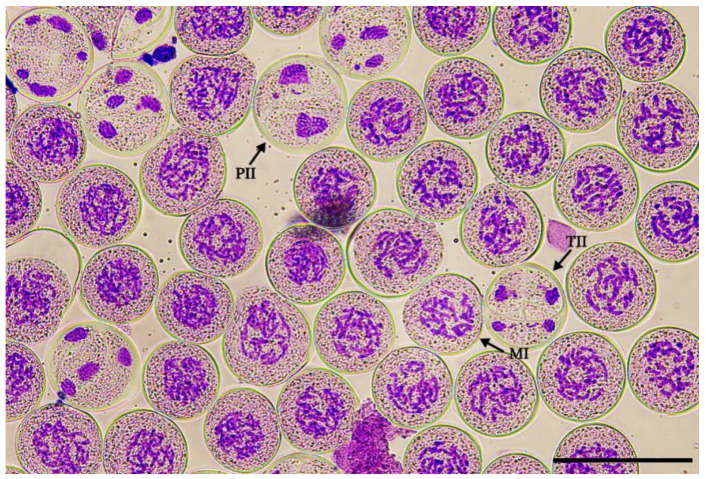
A mixture of meiotic stages in one flower bud. Arrows show M I (metaphase I), P II (Prophase II), and T II (Telophase II). (Bar = 100 μm).

In order to determine the optimal treatment time for the fertile pollen induction, the meiotic stages of PMCs of ‘Black Beauty’ were observed, and their corresponding traits with the length of the bud/anther were established (Figure 1, Table 1). The results show that meiosis had not started (Figure 1a) when the bud length was less than 28 mm, while the anther length was less than 16 mm. The buds ranging from 28 to 30 mm mainly exhibited prophase I (Figure 1b), with anthers ranged from 17 to 19 mm at this time. Mixed stages of meiosis were observed in buds ranging from 31 to 33 mm in the same flower bud (Figure 2), mainly including metaphase I (86.08%, Figure 1c), prophase II (2.32%, Figure 1f), and telophase II (11.60%, Figure 1i). When the length of the flower buds exceeded 33 mm, with the length of the anthers > 22 mm, most meiosis stages of PMCs entered the tetrad (Figure 1j). In previous studies on lily male gamete doubling, a higher proportion of 2n pollen can be obtained by treating buds at the meiotic stage of prophase I to metaphase I [10,13,19]. So, buds ranging from 28 to 31 mm were chosen as the induction material.

### 2.2. Morphological Characteristics after Treatment

**Figure 3:**
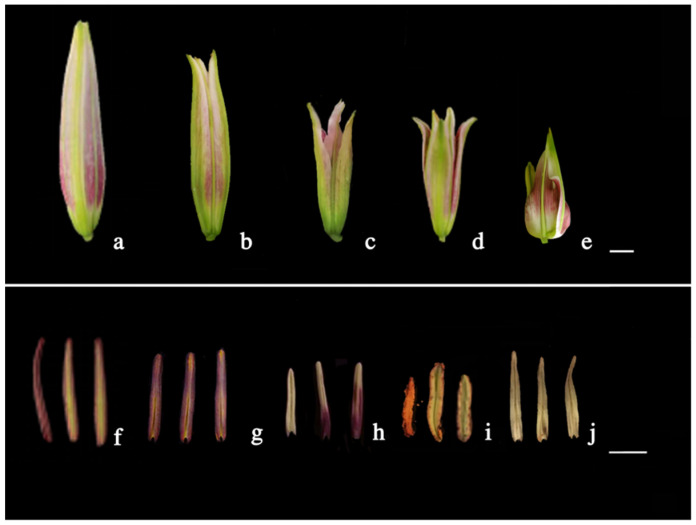
Flower buds and their corresponding anther morphology on the day of flowering for the different treatments and controls. (**a**,**f**) Control flower bud and anthers without any treatment. (**b**,**g**) Flower bud and anthers treated at a high temperature of 40 °C for 4 h. (**c**,**h**) Flower bud and anthers treated at a high temperature of 42 °C for 6 h (Reduced or no pollen where anthers turn white). (**d**,**i**) Flower bud and anthers treated with 0.01% concentration of oryzalin. (**e**,**j**) Flower bud and anthers treated with 0.01% concentration of colchicine (outer petals deciduous) (Bar = 10 mm).

Flower buds of all treatment groups were abnormally developed after colchicine and oryzalin induction compared with the sterile distilled water control (Figure 3). They showed flower bud deformity, while petals, filaments, ovaries, and styles exhibited shortening and hypertrophy. Anthers were also obviously abnormal. Some anthers treated with colchicine had no pollen and were shrunken and wilted. While pollen was available in all treatment groups of oryzalin, parts of the anthers were covered with yellow pollen (normal pollen is dark red) and their morphology was significantly shorter than that of normal anthers.

Unlike the chemical treatment group, the growth and development of some flower buds in the high-temperature treatment group were relatively normal, and the morphology and structure of flower buds were normal, but the powder dispersion was delayed for 2–3 d. Some buds were deformed; petals and ovaries were shortened in different degrees, and some anthers were discolored and exhibited reduced or no pollen (Figure 3).

### 2.3. Effect of High-Temperature Treatment on Fertile Pollen Induction

**Figure 4:**
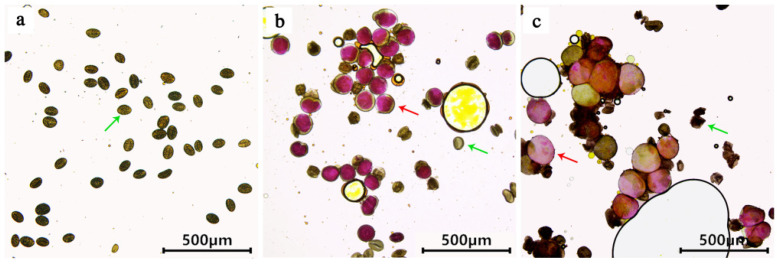
Comparison of sterile and fertile pollen from ‘Black Beauty’. (**a**) Control group. (**b**) Pollen grains obtained after 4 h treatment at 42 °C. (**c**) Pollen grains obtained after 6 h treatment at 42 °C. (Nonfertile pollen indicated by green arrows, and fertile pollen indicated by red arrows).

The six treatments were designed for the high-temperature induction, and all of them produced fertile pollen grains (Figure 4). The percentage of induced fertile pollen ranged from 0.19% to 36.70% (Table 2). Bud mortality was proportional to temperature and time.

The general linear model (GLM) univariate analysis of variance for fertile pollen induction showed that treatment temperature (F = 63.052, *p* < 0.001) and duration (F = 163.403, *p* < 0.001) significantly affected fertile pollen induction rates (Table 3). The least significant difference (LSD) test (α = 0.05) showed that the average incidence (%) of fertile pollen induced by the 42 °C and 44 °C treatments was significantly higher than 40 °C at a duration of 4 h, while there was no significant difference between 42 °C and 44 °C. The average incidence (%) of fertile pollen induced by various temperature treatments was not significantly different in the duration of 6 h. The average incidence (%) of fertile pollen induced by the 4 h treatment was significantly higher than the mean incidence (%) at 6 h when the treatment temperature was 42 °C and 44 °C. Therefore, taking together the percentage induction of fertile pollen and bud mortality, the best treatment was to treat the buds using a high temperature of 42 °C for 4 h. Under these conditions, the highest induction rate (%) of fertile pollen was 36.64% (Figure 4, Table 2). No fertile pollen was found in the nontreated control group.

The pollen diameters of all experimental groups were statistically analyzed. The average diameter of naturally produced control pollen was 58.78 μm, while fertile pollen with a treatment duration of 4 h and 6 h was 107.88 μm and 138.64 μm, respectively, which means it was 1.83- and 2.35-fold larger than that of the control pollen (Table 2). GLM univariate analysis of the average diameter of the control sterile pollen and high-temperature induced fertile pollen showed that the treatment temperatures (F = 0.106, *p* > 0.05) were not statistically different, while the durations (F = 10.776, *p* < 0.01) significantly affected the mean pollen diameter (Table 4). The LSD test (α = 0.05) showed that the average diameter of pollen induced by the 6 h treatment was significantly larger than that of the 4 h treatment, while the average diameter of pollen induced by the 4 h treatment was also significantly higher than that of the control. The average pollen diameter induced by the different treatment temperatures was not significantly different, but was significantly higher than the control pollen grains (Figure 4).

### 2.4. Effects of Chemical Treatments on Fertile Pollen Induction

Two chemicals, colchicine and oryzalin, were used for the induction of fertile pollen. The results show that no fertile pollen was obtained after colchicine treatments (Table 5), potentially because they were highly toxic to ‘Black Beauty’ or no suitable treatment concentration was found.

The effect of oryzalin on the induction of fertile pollen is shown in Table 6, and the average percentages of oryzalin-induced fertile pollen ranged from 0 to 15.39%. The LSD test (α = 0.05) showed that there were no significant differences between concentrations of 0.01% and 0.015% in the mean induction rate of fertile pollen, but was significantly lower than the concentration of 0.005%.

No fertile pollen production was detected in the distilled water as the control. In addition, the flower bud mortality also increased gradually with increasing treatment concentrations due to the toxic effects of colchicine and oryzalin (Table 5 and Table 6).

### 2.5. Polyploid Production by Crossing with Fertile Pollen

Fertile pollen induced by a high temperature (duration of 4 h) was used to hybridize with two OT cultivars and a Trumpet lily (T) as maternal parents. A total of 267 seeds with embryos were obtained, and the highest embryonic rate of 4.52% was obtained with ‘Mister Cas’ (Table 7). The pollen without treatment was used as the control, and no embryonated seeds were obtained. After inoculation of the embryonic seeds in a sterile system, the germination rate of seedlings was 26.28% for the cross-combination with ‘Mister Cas’ as the maternal parent, while it was 28.57% with *L. regale*.

## 3. Discussion

Interspecific distant hybrid is an important tool and breeding trend for breeding new varieties of lilies. The F_1_ distant hybrids usually exhibit intermediate traits of both parents and are usually highly sterile due to the large distance in genetic composition, limiting the further improvement and progressive breeding of lilies [2,3]. Therefore, restoring the fertility of the F_1_ generation of distant hybrids is a critical issue in breeding new varieties of lilies.

Mitotic polyploidization and meiotic polyploidization are common methods of restoring fertility in F_1_ distant hybrids, of which mitotic polyploidization has the problem of long breeding cycles and less variation in backcrossed progeny. Meiotic polyploidization, on the other hand, can produce fertile pollen with a rich genetic composition and promote homologous recombination, resulting in more variation in the progeny, allowing the selection of new varieties with excellent traits [4,5].

### 3.1. Feasibility of High-Temperature Induction of Lilies

A suitable induction method is essential for the 2n gamete induction. Chemicals, such as colchicine, are now commonly used in plants to induce 2n gametes. As an antimicrotubule and antimitotic agent, colchicine prevents microtubule formation by binding to tubulins, leading to cytokinesis failure and achieving chromosome nonreduction [20,21]. The 2n pollen production rate was highest at 28.71% after 6 h of treatment with 0.5% colchicine in *Eucalyptus* [22]. In *Paeonia lactiflora*, the maximum incidence of 2n pollen induction (%) was 47.39% after two injections with 0.4% colchicine [23]. Other herbicides, such as oryzalin, pronamide, and trifluralin, are also widely used as alternatives to colchicine in the doubling field, showing a high affinity to plant tubulin and therefore can be used in lower concentrations [24]. N_2_O also induces polyploidization by inhibiting microtubule polymerization [25] and can be used in lily 2n gamete induction. However, the equipment and its operation are somewhat specialized and dangerous and are not easy to handle.

High temperatures have also been shown to induce the production of 2n gametes in plants; however, the effect of the interactions between different plant genotypes and environmental factors on their production has not been determined [11]. It has been successful in *P. tomentosa* [14] and *H. brasiliensis* [15], but has rarely been reported in lilies. Using F_1_ sterile hybrids as material, the experimental results showed substantial differences in 2n pollen induction rates in different environments. Three out of the four Oriental × Asiatic (OA) lily hybrids could produce viable 2n pollen in a greenhouse with fluctuating temperatures [17]. In addition, planting triploid sterile hybrids of OT lily and Longiflorum × Asiatic (LA) lily in a growth chamber at 30 °C for 7 d also yielded viable pollen, with up to 8.4% pollen germination [16]. In this study, self-made multiline heating equipment was used, and three temperatures and two treatment times were chosen to investigate the suitability of high-temperature induction of lily 2n gametes. The experimental results show that 42 °C treatment for 4 h was the best treatment combination, inducing a maximum of 36.64% of fertile pollen, much higher than the maximum of 15.39% of 2n pollen induction with oryzalin.

### 3.2. Speculation on the Effect of Different Temperatures on Pollen Ploidy

The pollen diameter can be used to estimate sporophytic ploidy levels in many species [23,26,27]. In the present study, the high-temperature treatment produced a wide range of pollen diameters (from 51.37 μm to 212.30 μm), even for the same treatment combination, indicating a wide range of ploidy. The average diameter of the control pollen was 58.78 μm, while the average diameter of the fertile pollen treated for 4 h and 6 h was 107.88 μm and 138.64 μm, respectively, which is 1.83 and 2.35 times the average diameter of the control pollen, while the average diameter of the fertile pollen for 6 h was 1.28 times larger than that of 4 h. This could imply that the high-temperature treatment produced oversized pollen grains that were 3n or 4n pollen grains, with a greater proportion of large pollen induced by the prolonged 6 h treatment.

Environmental factors, such as temperature, are one of the most important factors that influence or determine the duration of meiosis [28]. In the present study, the fertile pollen rates obtained from high-temperature induction were statistically significantly different between the different treatment combinations, ranging from 0.19% (6 h at 44 °C) to 36.70% (4 h at 44 °C), with the 2n pollen rate being strongly affected by both treatment temperature and duration. However, we know little about the mechanism of chromosome doubling in lilies caused by high temperatures. Based on relevant studies of other plants, it is speculated that the formation of the spindle during meiosis prophase I and metaphase I was inhibited by high-temperature treatment, causing chromosomes not to meioticize [14]. Alternatively, high-temperature treatment may cause abnormal spindle structure, forming parallel, fused, or tripolar spindles, resulting in the production of 2n gametes [29]. The relevant research work in this area needs to be advanced.

High temperatures may also accelerate the meiotic process, resulting in the failure of two meiotic divisions. In *L. candidum* [30] and *L. longiflorum* [31], it takes only 24 and 48 h from metaphase I to telophase II, respectively. In this study, the meiosis of PMCs only occurred in the buds measuring 28–33 mm in length, which might imply a short duration of meiosis in the PMCs of ‘Black Beauty’. At the same time, the high-temperature treatment may have further accelerated the meiotic process in ‘Black Beauty’, thus shortening the meiotic time. Misorientation of the thermally induced spindle in telophase II can also lead to dyad or triad formation [32], implying that two meiotic failures may have occurred in our study (especially for the 6 h duration), resulting in oversized pollen grains.

The formation of walls of PMCs in lilies may also be disturbed by high-temperature treatments. In the present study, we observed a variety of forms of conjoined pollen, including one large with one small, two of the same size, and two large with one small, suggesting abnormal pollen wall formation. The organization or stability of microtubules in *Nicotiana tabacum* mitotic cells would be modified by high-temperature treatment, resulting in defective cell wall formation [33], and this would lead to the formation of n, 2n, 3n, and 4n pollen. High temperatures can also affect the normal function of the anthers, including tapetum, which can produce callase. The lack or temporary reduction in callase enzyme activity can affect the formation of the microspore cell wall and ultimately the level of fertility [32]. The discoloration of the anther wall and the reduction in pollen or even being pollen-free in this study indicated that the anther structure had been damaged by high temperatures, which can also supports the hypothesis.

### 3.3. Selection of Cross Parents

There are several problems in the selection of maternal parents. On the one hand, the affinity is unknown, and many crosses would be required to find suitable females. Because OT lilies are mostly triploid and the male gametes are highly sterile, they were mostly used as maternal parents in previous studies, while the combinations that used them as paternal parents were mostly unfruitful. Cao et al. found that two out of three crosses with *L. regale* as the paternal parents and OT lily as the maternal parents produced embryonic seeds, with a maximum embryo rate of 8.96% [34]. In the study by Zhang et al., among nine OT × OT cross combinations, only two combinations with ‘Saltarello’ as the maternal parent yielded embryonic seeds [35]. In Cao et al.’s study, sixteen of twenty-four OT × O cross combinations could bear fruit, but four O × OT backcross combinations and six OT × OT self-cross combinations failed [36]. This suggests that crosses with OT lilies as the paternal parents are relatively difficult, and therefore the selection of suitable parents to avoid pre- and postfertilization barriers is important for the success of crosses [37]. So, in the present study, *L. regale* and ‘Saltarello’, which have been shown to be suitable for maternal parents in previous studies, were selected. On the other hand, 2n pollen is less viable, and the pollen tubes germinate slowly or fail to grow to a corresponding length [38,39], and there are no relevant studies on how low-temperature storage related to induced 2n pollen. To avoid the possible effects of reduced pollen activity due to low-temperature storage, females with high fertility and similar flowering periods were selected for crosses whenever possible. The flowering period of ‘Black Beauty’ is different from that of its parents; therefore, no backcrossing was performed. Of all six cross combinations, only two of the crosses yielded embryonic seeds with a maximum embryonic rate of 4.52%, which was consistent with previous studies. Many crosses still need to be continued in the future to further explore the affinity of parents to breed new cultivars of OT lilies.

## 4. Materials and Methods

### 4.1. Plant Materials

In this study, the sterile OT F_1_ hybrid ‘Black Beauty’, selected from *L. rubrum* (speciosum) and *L. henry,* was used to induce fertile pollen. The fertile pollen induced by high temperatures (duration of 4 h) was crossed with three cultivars of OT lily, two of Trumpet lily (T), and one of Oriental lily (O) as maternal parents. All plants were planted in the germplasm resources nursery of Beijing Forestry University. Due to the late flowering of ‘Black Beauty’, which had an impact on subsequent crosses, and to avoid the decrease of pollen activity that may be caused by pollen refrigeration, bulbs were put into cold storage in advance and planted on 19 January to achieve an earlier flowering date and facilitate crosses.

### 4.2. Determination of PMC Meiotic Stage

The length of lily buds is highly correlated with the meiosis stage [40]. In order to determine the suitable stage for treatments, anthers of different lengths of flower buds (from 5 mm to 40 mm) were taken; three to five anthers per bud were taken for observation, and the meiotic stages of 300–500 PMCs per bud were assessed using a SDPTOP CX40 microscope (Zhejiang, China). The anthers were removed and cut into 1 mm sections and placed on slides. We removed unnecessary debris of the anther wall under an anatomical microscope and spread the PMCs on the slide [41]. A total of 16 μL carbol fuchsin solution (Solarbio, Beijing, China) was swiftly added and gently mixed with PMCs. The meiotic stages of PMCs were observed from interphase to tetrad phase. Then, the correspondence between the meiosis stage of PMCs and the length of flower buds was established.

### 4.3. Induction of Fertile Pollen by High-Temperature Treatment

Based on the above results, flower buds during the prophase I–metaphase I were chosen for treatments. They were treated separately using our self-made multiwire heating equipment (unpublished); each wire was connected to a heating bag, and each heating bag holds only one bud, so that each bud can be treated individually for precise temperatures without causing heat stress to the whole plant. Different heating temperatures (40 °C, 42 °C, and 44 °C) and heating durations (4 h and 6 h) were set for a total of six treatment combinations, and the untreated buds of the same length on the same day served as the control group. Five buds of each treatment or control group were treated with three replicates.

### 4.4. Induction of Fertile Pollen by Chemicals

To compare the effects of high-temperature and chemical treatments on the induction of fertile gametes, different concentrations of colchicine (0.05%, 0.1%, 0.2%, 0.3%, 0.4%, and 0.5%) and oryzalin (0.005%, 0.01%, 0.015%, 0.02%, and 0.025%) were injected into the same-stage flower buds with a 10 µL microsyringe before 11 am. Ten buds of each treatment or control group were treated with three replicates.

### 4.5. Testing Pollen Fertility

Anthers of different experimental groups were collected before anthesis, dried in a cool, clean place on a Sulfuric acid paper bag separately, collected in centrifuge tubes with silica gel, and stored in a refrigerator at 4 °C. Alexander staining (Solarbio, Beijing, China) was used to check the pollen viability [42]. Round pollen grains stained magenta red were considered fertile, while unstained oval pollen grains were considered sterile. Over 450 pollen grains from each flower bud of experimental groups were counted, and photographs were taken using a SDPTOP CX40 microscope. The diameters of fertile pollen grains stained magenta red and sterile control pollen grains were measured separately using the software ImageView at the same time.

### 4.6. Crossing Experiment

In our study, Trumpet lily *L. regale*, *L. sargentiae*, and Oriental lily ‘Entertainer’ and OT hybrids ‘Mister Cas’, ‘Beverly Dreams’, and ‘Saltarello’ were used as maternal parents and crossed with high-temperature-induced pollen grains with 4 h duration that confirmed to be fertile. The female parents were emasculated before anthesis. When the stigmas began to secrete mucus, pollination was performed, and the stigmas were wrapped in tinfoil to prevent pollution with external pollen. Two months after pollination, fruits were collected, and the percentage of embryonic seeds was counted (percentage of embryonic seeds% = number of seeds with embryos/total number of seeds × 100%). Seeds with embryos were all inoculated on MS medium with 40 g sucrose, 2mL6-BA, and 0.1 mg NAA per liter.

### 4.7. Statistical Analysis

Fertile pollen induction rates and average fertile pollen diameters were analyzed using GLM univariate for high temperatures to reveal differences between temperature and duration in high-temperature treatment. Data were transformed for the incidence of induced fertile pollen (1/p) before analysis of variance was performed to account for heterogeneous variance. LSD was used for multiple comparison tests (*p* < 0.05). The statistical analyses were all carried out using SPSS software (SPSS for Windows, Version 26, SPSS, Chicago, IL, USA).

## 5. Conclusions

In this study, ‘Black Beauty’ lily buds with PMCs at the meiotic stage of prophase I to metaphase I were treated with high temperatures using multiwire heating equipment, chemical injections (including colchicine and oryzalin), and no treatment as the control. The results show that high-temperature treatment could induce fertile pollen, and the highest rate of fertile pollen produced (36.64%) was significantly higher than that of oryzalin (15.39%), while no fertile pollen was obtained in the colchicine and untreated groups. The treatment temperature and duration significantly influenced the fertile pollen induction rate. Excessive temperature and duration of treatment damaged the pollen and external petal development of the buds, resulting in a reduced yield and increased bud drop. In particular, the average diameter of the 6 h treatment group was also larger than that of the 4 h treatment group, which may be related to the failure of the two meiotic divisions and the disruption of the pollen wall formation due to the long duration. By crossing the fertile pollen produced by induction, embryonic seeds were obtained, demonstrating that high temperatures might be a worthwhile method in the field of lily distant hybrid breeding and polyploid breeding.

## Figures and Tables

**Table 1:** Correlation between bud/anther lengths and meiotic stage in PMCs of ‘Black Beauty’.

Bud Length (mm)	Anther Length (mm)	Meiotic Stage
<28	<16	Interphase
28–30	17–19	Prophase I
31–33	20–21	Metaphase I–Telophase II
>33	>22	Tetrad

**Table 2:** Effects of different high temperatures and durations on buds and fertile pollens of ‘Black Beauty’.

Temperature (°C)	Duration (h)	No. of Buds Treated	Flower Bud Mortality (%)	Fertile Pollen (%)	Average Diameter of Fertile Pollen (μm)
40	4	15	0.00	2.09 ± 1.38	106.53 ± 13.34
	6	15	20.00	1.55 ± 1.54	139.81 ± 14.51
42	4	15	40.00	36.64 ± 1.75	112.50 ± 27.01
	6	15	53.33	2.17 ± 2.01	139.53 ± 12.67
44	4	15	86.67	36.70 ± 3.77	104.62 ± 1.95
	6	15	66.67	0.19 ± 2.38	136.59 ± 38.75
Control	0	15	15	0.00 ± 0.00	58.78 ± 0.93 (Sterile pollen)

**Table 3:** GLM univariate analysis of the incidence of high-temperature-induced fertile pollen.

Variance	SS	df	MS	F Value	P
Temperature	3575.207	2	1787.604	63.052	0.000
Duration	4632.667	1	4632.667	163.403	0.000
Error	1247.453	44	28.351		
Total	16,150.767	50			

**Table 4:** GLM univariate analysis of pollen grain diameters of control and high-temperature treatments.

Variance	SS	df	MS	F Value	P
Temperature	71.983	2	35.991	0.106	0.900
Duration	3649.542	1	3649.542	10.776	0.007
Error	4064.043	12	338.670		
Total	262,693.646	19			

**Table 5:** Effects of colchicine with different concentrations on fertile pollen induction for ‘Black Beauty’.

Induction Method	Colchicine Concentration (%)	Fertile Pollen (%)	Flower Bud Mortality (%)
Colchicine	0.05	0.00 ± 0.00	43.33
	0.1	0.00 ± 0.00	30.00
	0.2	0.00 ± 0.00	33.33
	0.3	0.00 ± 0.00	76.67
	0.4	0.00 ± 0.00	80.00
	0.5	0.00 ± 0.00	83.33
Control	0.0	0.00 ± 0.00	0.00

**Table 6:** Effects of oryzalin with different concentrations on fertile pollen induction for ‘Black Beauty’.

**Induction Method**	**Oryzalin** **Concentration (%)**	**Fertile Pollen (%)**	**Flower Bud Mortality** **(%)**
Oryzalin	0.005	15.39 ± 8.05	36.67
	0.01	6.89 ± 5.70	56.67
	0.015	1.80 ± 1.74	66.67
	0.02	0.00 ± 0.00	80.00
	0.025	0.00 ± 0.00	60.00
Control	0.00	0.00 ± 0.00	0.00

**Table 7:** Statistics of cross-pollination results with induced fertile pollen.

Type	Maternal Parents	No. of Crossings	Ovaries Developed (%)	No. of Seeds with Embryos	Seeds with Embryos (%)	No. of Seedlings
OT	‘Mister Cas’	57	77.19	274	4.52	72
	‘Beverly Dreams’	20	0.00	0	0.00	-
	‘Saltarello’	10	0.00	0	0.00	-
O	‘Entertainer’	15	0.00	0	0.00	-
T	*L. regale*	10	10.00	7	1.72	2
	*L* *. sargentiae*	10	0.00	0	0.00	-

## Data Availability

Not applicable.
